# Candidates’ Examination, State of Ohio

**Published:** 1895-11

**Authors:** 


### CANDIDATES' EXAMINATION, STATE OF OHIO.

The following are the questions asked by the Ohio State Board of Veterinary Examiners, July 2, 1895, at Columbus:

1. Describe the tarsal joint of the horse. 2. Describe the digestive canal of a cow from mouth to anus. 3. Describe the structure of the liver. 4. Name and describe the cerebral nerves. 5. Describe the process of digestion in a ruminant, and state how rumination is effected. 6. Describe the circulation of the blood in the fœtus. 7. Name and describe three poisonous umbelliferæ, and state where they grow, and what effect they have upon herbivorous animals. 8. What is an element? Of what elements is the animal organism composed? 9. What is an organic and what is an inorganic compound? 10. If calomel and common salt constitute the ingredients of the same medicine, what will happen, and why? 11. How would you differentiate a cataract from amaurosis? 12. What infirmities of the extremities would you look for in examining a horse for soundness? 13. Give the life-history of tænia cœnurus. 14. Give a definition of the morbid process usually called "inflammation." 15. State and describe the characteristic morbid changes in contagious pleuro-pneumonia of cattle, *i. e.*, those of the greatest diagnostic importance. 16. Describe symptoms and morbid changes of rabies in dogs. 17. Describe and explain the most frequent predisposing cause of colic in horses. 18. Describe the operation of castrating a horse with covered testicle. 19. Describe the disease known as spavin. Give the characteristic symptoms, especially such as are of diagnostic value, and describe the morbid changes of the various stages. 20. If in a case of obstetrics you should find all four feet of the fœtus in the os of the uterus, how would you proceed to deliver the mare? 21. How would you proceed in a case of prolapsus of the uterus in a cow if the prolapsus is a complete one? 22. How would you shoe a horse with pumiced hoofs, and how one that has a suppurating corn? 23. What is the effect of digitalis upon a horse? What constitutes a safe dose? And how often may it be repeated? Explain the principal effect. How would you diagnosticate, and, after the diagnosis has been made, treat an impaction of the third stomach in a cow? 24. If at post-mortem examination of a horse, which you have not seen during life, you find a bucketful of clear serum in the chest, what, in your opinion, is the shortest possible time required for such a large effusion, or how long must it have existed? 25. If you are called to see a lot of sick hogs, and you find that they are running around in a circle, and evidently suffering from an affection of the brain, all acting in the same manner, what, in your opinion, has probably happened, or what conclusion, naturally, would you come to concerning the cause?

---

THOUGH the amended Act regulating the State Veterinary Sanitary Service in Missouri was directed to enable the State Board of Health to deal with bovine tuberculosis in some practical way, as yet we can learn of nothing done toward accomplishing a satisfactory solution of this important need of her people. Will the Board make public the cause of delay?

